# Effect of Plasma Level of Vitamin D on Postoperative Atrial Fibrillation in Patients Undergoing Isolated Coronary Artery Bypass Grafting

**DOI:** 10.21470/1678-9741-2017-0214

**Published:** 2018

**Authors:** Kadir Kaan Özsin, Umut Serhat Sanrı, Faruk Toktaş, Nail Kahraman, Şenol Yavuz

**Affiliations:** 1 Department of Cardiovascular Surgery, Bursa Yuksek Ihtisas Training and Research Hospital, University of Health Sciences, Yıldırım/Bursa, Turkey.

**Keywords:** Vitamin D, Postoperative Period, Atrial Fibrillation, Coronary Artery Bypass

## Abstract

**Objective:**

Postoperative atrial fibrillation (PoAF) is a common complication after coronary artery bypass grafting (CABG). The aim of the present study was to evaluate the association between development of PoAF and vitamin D levels in patients undergoing isolated CABG.

**Methods:**

This prospective randomized clinical trial was conducted on the patients with isolated CABG. The study was terminated when 50 patients in both PoAF(+) group and PoAF(-) group were reached. Development of AF until discharge period was assessed. Vitamin D level was measured immediately after AF; it was measured on the discharge day for the patients without PoAF. Predictive values of the independent variables were measured for the development of PoAF.

**Results:**

The groups were separated as PoAF(-) group (66% male, mean age 58.18±10.98 years) and PoAF(+) group (74% male, mean age 61.94±10.88 years). 25(OH) vitamin D level (OR=0.855, 95% CI: 0.780-0.938, *P*=0.001) and > 65 years (OR=3.525, 95% CI: 1.310-9.483, *P*=0.013) were identified as an independent predictor of postoperative AF after CABG surgery in multivariate analysis. The cut-off level for 25(OH) vitamin D level in receiver-operating characteristic curve analysis was determined as 7.65 with sensitivity of 60% and specificity of 64% for predicting PoAF (area under the curve: 0.679, *P*=0.002).

**Conclusion:**

Vitamin D level is considered an independent predictor for development of PoAF. Lower vitamin D levels may be one of the reasons for PoAF.

**Table:** 

Abbreviations, acronyms & symbols	
25(OH) vitamin D	= 25-hydroxy vitamin D
ACE	= Angiotensin-converting enzyme
AF	= Atrial fibrillation
ARB	= Angiotensin receptor blocker
CABG	= Coronary artery bypass grafting
CI	= Confidence interval
COPD	= Chronic obstructive pulmonary disease
CPB	= Cardiopulmonary bypass
ECG	= Electrocardiography
ICU	= Intensive care unit
OR	= Odds ratio
PoAF	= Postoperative atrial fibrillation
RAAS	= Renin-angiotensin-aldosterone system
ROC	= Receiver-operating characteristic curve

## INTRODUCTION

Postoperative atrial fibrillation (PoAF) is a common complication after coronary artery bypass grafting (CABG), affecting about 20% to 35% of all patients in the early postoperative period^[^^1^^]^. PoAF is commonly associated with hemodynamic instability, thromboembolic events, increase in early and late mortality rates, heart failure progression and increased duration of hospitalization^[^^1^^]^.

Vitamin D exists in two forms: D2 (ergocalciferol) and D3 (cholecalciferol). Vitamin D is converted into calcidiol and calcitriol, respectively in the liver and kidney; and acts on specific target tissues via vitamin D receptors^[^^2^^]^. Vitamin D receptors were found in other extra-osseous tissues, as well as all major cardiovascular cell types, including cardiomyocytes, vascular smooth muscle cells, endothelial cells, the brain, pancreatic beta-cells, skeletal muscles, breast, prostate, colon, macrophages, and skin, exerting several pleiotropic effects, and their expression decreases with age^[^^3^^]^.

Deficiency of vitamin D may cause a number of diseases. Some of these diseases include type 2 diabetes mellitus, metabolic syndrome, obesity, hypertension, and some cardiovascular disease^[^^4^^]^. Several pathophysiological mechanisms were suggested for the association between vitamin D deficiency and atrial fibrillation (AF). One of the most important mechanisms is the activation of the renin-angiotensin-aldosterone system (RAAS), as it is responsible for both structural and electrical remodeling of the atrium. Vitamin D negatively affects the RAAS, and it has antioxidant effects that reduce oxygen free radicals in the atria which are associated with inflammation and the production of proarrhythmic materials^[^^5^^]^.

Presence of vitamin D receptor in extra-osseous tissues; and the link between vitamin D and the RAAS may be shown relationship between vitamin D and AF risk factors. The aim of this study was to determined the relationship between vitamin D levels and PoAF in patients undergoing isolated CABG.

## METHODS

### The Patients

This prospective randomized clinical trial was conducted on the patients with isolated CABG diagnosed at of Cardiovascular Surgery Department within Bursa Yuksek Ihtisas Training and Research Hospital, Bursa, Turkey, between November 2016 and May 2017. The study was approved by the local institutional Ethical Committee of the University of Health Sciences. All procedures were performed in accordance with the Declaration of Helsinki.

Inclusion criteria was isolated CABG. The exclusion criterias were having preoperative AF or flutter, previous treatment with amiodarone, presence of valvular heart disease, chronic obstructive pulmonary disease (COPD), prolonged intensive care unit (ICU) stay, patients who underwent more than one cardiac surgery, bleeding revision, chronic renal failure. Patients who underwent isolated CABG were followed until the day of discharge from the hospital. Patients who developed AF during the this period were included in the POAF(+) group, and patients without AF were enrolled in the PoAF(-) group. The study was terminated when 50 patients were reached in both groups.

The data of PoAF(+) group (74% male, mean age 61.94±10.88 years) and PoAF(-) group (66% male, mean age 58.18±10.98 years) are shown in Table 1.

**Table 1:** Demographic features of the patients.

	PoAF(+) group (n=50)	PoAF(-) group (n=50)	*P* value*
Age (years)	61.94±10.88	58.18±10.98	0.089
Age ≥ 65 years, n (%)	22 (44)	12 (24)	0.035
Male gender, n (%)	37 (74)	33 (66)	0.383
Hypertension, n (%)	26 (52)	22 (44)	0.423
Diabetes mellitus, n (%)	23 (46)	21 (42)	0.687
Beta-blocker therapy, n (%)	38 (76)	32 (64)	0.190
Statin therapy, n (%)	31 (62)	29 (58)	0.683
ACE-I/ARB therapy, n (%)	22 (44)	20 (40)	0.685
BSA	1.85±0.16	1.87±0.16	0.719
BMI	27.69±4.48	27.56±4.06	0.881

* Student's t test; Pearson Chi-square

ACE-I=angiotensin-converting enzyme inhibitor; ARB=angiotensin-receptor blocker; BMI=body mass index; BSA=body surface area; PoAF=postoperative atrial fibrillation

All data were recorded as age, gender, history of hypertension, diabetes mellitus, preoperative drug use [beta-blockers, statins, angiotensin-converting enzyme (ACE) or angiotensin receptor blocker (ARB) inhibitors], ejection fraction, left atrial diameter, body mass index, body surface area, aortic cross clamp time, cardiopulmonary bypass time. Laboratory parameters were also studied from venous blood sample before the surgery except for 25-OH vitamin D levels. In the PoAF(+) group, the level of vitamin D was measured immediately after development AF. In the group of PoAF (-), the level of vitamin D was measured on the day patients were discharged from the hospital.

The inactive vitamin D precursors are first exposed to 25-hydroxylation in the liver to form 25-hydroxy vitamin D [(25(OH) vitamin D]. This is the actual circulating form of vitamin D and therefore is usually considered as a circulating biomarker for vitamin D status^[^^6^^]^.

The 25-OH vitamin D levels were measured through Architect 25-OH vitamin D- Reagent Kit (Abbott, Diagnostic Division Lisnamuck, Longford, Ireland). Reference ranges of the 25-OH vitamin D Kit used were 6.2 to 45.5 ng/mL for winter season and 7 to 53.2 ng/mL for summer season.

### Diagnosis of PoAF

The patients were monitored in ICU with continuous heart rhythm and invasive blood pressure monitoring. In addition, a 12-lead electrocardiography (ECG) record was also obtained daily in the ICU. Patients were monitored continuously by five-lead telemetry in the regular ward. When the patients complained about palpitation, dyspnea and angina, 12-lead ECG was taken. AF was confirmed by 12-lead ECG. Postoperative AF was described as irregular, fast oscillations or fibrillatory waves instead of regular *P* waves at ECG. An AF episode longer than 5 minutes was accepted as PoAF. Standard medical cardioversion treatment was conducted with amiodarone (5 mg/kg) for 30 minutes, followed by 900 mg/day.

### Statistical Analysis

Statistical analysis data were analyzed with the Statistical Package for the Social Sciences (IBM SPSS Statistic Inc. version 21.0, Chicago, IL, USA). Continuous and ordinal variables were expressed as mean ± standard deviation and nominal variables were expressed as frequency and percentage. Kolmogorov-Smirnov test and Shapiro-Wilk tests of normality were used to identify distribution of variables. Student's t test was used to compare two groups for continuous variables with normal distribution. Chi Square test was used to compare two groups for nominal variables. Mann-Whitney U test was used to compare two groups for continuous variables without normal distribution. For all tests, a *P* value of <0.05 was considered statistically significant. The relationship between the preoperative independent variables and the development of postoperative AF was evaluated by a logistic regression analysis. Receiver-operating characteristic (ROC) curve was applied for the prediction of PoAF development in patients undergoing isolated CABG and the area under the curve was calculated for vitamin D levels.

## RESULTS

In the present study, 50 patients were enrolled into each group. The demographic and clinical characteristics of the participants are summarized in Table 1. Being over 65 years of age was evaluated as a different parameter. Patients with PoAF were similar to patients without PoAF in regards to demographic properties, in generally. But, in terms of in patients with age > 65 years was statistically significant difference between two groups (*P*=0.035) (Table l).

The comparison of laboratory and operative parameters are shown in Table 2. Significant difference only was observed between two groups in terms of 25(OH) vitamin D levels. Twenty-five(OH) vitamin D level were significantly lower in the PoAF group (*P*=0.002) (Table 2). However, all other parameters were not significantly different between the groups (Table 2).

**Table 2:** Laboratory and operative variables.

	PoAF group (n=50)	PoAF(-) group (n=50)	*P* value*
Hematocrit (%)	39.37±5.53	41.16±4.69	0.244
White blood cell (10^3^/µL)	9.18±2.14	9.55±2.87	0.733
Platelet (10^3^/µL)	268.98±91.2	237.56±54.69	0.126
Red cell distribution width (%)	13.76±1.13	13.98±1.25	0.456
Mean platelet volume (fL)	8.91±0.98	8.66±0.86	0.208
BUN (mg/dL)	18.04±7.71	17.78±7.39	0.803
Creatinine (mg/dL)	0.88±0.21	0.89±0.32	0.545
Na (mEq/L)	138.3±3.07	138.64±2.46	0.534
K (mEq/L)	4.17±0.42	4.14±0.6	0.673
Ca (mg/dL)	9.09±0.42	9.17±0.51	0.269
Mg (mg/dL)	1.91±0.15	1.9±0.22	0.816
Free T3 (pg/mL)	2.94±0.45	2.95±0.44	0.959
Free T4 (ng/dL)	1.11±0.19	1.17±0.21	0.166
TSH (IU/mL)	1.96±1.19	2.92±4.72	0.608
C reactive protein (mg/dL)	14.73±23.40	12.7±19.09	0.871
Total cholesterol (mg/dl)	202.5±33.41	197.92±39.96	0.536
LDL-C (mg/dL)	128.11±29.44	119.37±33.57	0.596
HDL-C (mg/dL)	43.38±8.36	41.55±8.43	0.316
TG (mg/dL)	153.38±65.66	184.44±111.41	0.237
25(OH) vitamin D (ng/mL)	7.49±3.81	12.13±7.98	0.002
Ejection fraction (%)	51.14±9.09	50.20±9.42	0.648
Left atrium diameter (mm)	38.40±4.22	38.08±4.15	0.928
ACC time (min)	58.32±14.99	57.04±14.38	0.473
CPB time (min)	88.04±20.99	86.72±1947	0.745

* Student's-t test; Mann-Whitney U test

25(OH) vitamin D=25-hydroxy vitamin D; ACC=aortic cross clamp; BUN=Blood urea nitrogen; CPB=cardiopulmonary bypass; HDL-C=high density lipoprotein cholesterol; LDL-C=low density lipoprotein cholesterol; PoAF=postoperative atrial fibrillation; T3=triiodothyronine; T4=thyroxine; TG=triglyceride; TSH=thyroid-stimulating hormone

**Fig. 1:**
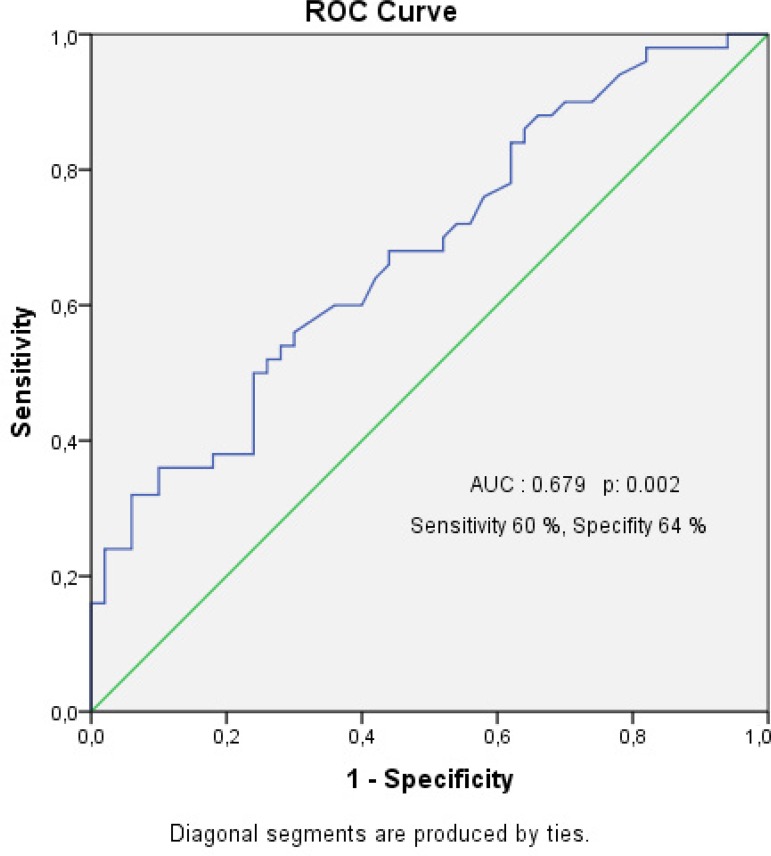
Receiver operation characteristic (ROC) curve and the area under the curve (AUC) for vitamin D levels for predicting PoAF.

Risk factors related to the development of PoAF were included univariate logistic regression analysis. In univariate logistic regression analysis, the PoAF was significantly correlated with 25(OH) vitamin D level (OR [odds ratio]=0.867, 95% CI [Confidence interval]: 0.793-0.949, *P*=0.002) and age > 65 years (OR=0.402, 95% CI: 0.171-.946, *P*=0.037), but was not correlated with age (OR=1.032, 95% CI: 0.995-1.071, *P*=0.091), hypertension (OR=1.379, 95% CI: 0.628-3.029, *P*=0.424), diabetes mellitus (OR=1.176, 95% CI: 0.534-2.593, *P*=0.687), ejection fraction (OR=1.011, 95% CI: 0.969-1.055, *P*=0.609), left atrium diameter (OR=1.019, 95% CI: 0.927-1.120, *P*=0.700) and cardiopulmonary bypass (CPB) time (OR=1.003, 95% CI: 0.984-1.023, *P*=0.742) (Table 3). Similar to the results in the univariate analysis, 25(OH) vitamin D level (OR=0.855, 95% CI: 0.780-0.938, *P*=0.001) and age > 65 years (OR=3.525, 95% CI: 1.310-9.483, *P*=0.013) were identified as an independent predictor of postoperative AF after CABG surgery in multivariate analysis (Table 3). Additionally, it was determined a cut-off level of 7.65 for 25(OH) vitamin D level for predicting PoAF with a sensitivity of 60% and a specificity of 64%, in ROC curve analysis (area under the curve: 0.679, 95% CI: 0.576-0.783, *P*=0.002) (Figure 1).

**Table 3:** Binary logistic regression analysis to identify predictors of PoAF.

	Univariate Analysis			Multivariate analysis		
Variables	*P*	Exp(B) Odds ratio	95% C.I. Lower Upper	*P*	Exp(B) Odds ratio	95% C.I. Lower Upper
Age	0.091	1.032	0.995-1.071			
Age ≥ 65 years	0.037	0.402	0.171 - .946	0.013	3.525	1.310-9.483
HT	0.424	1.379	0.628-3.029			
DM	0.687	1.176	0.534-2.593			
EF	0.609	1.011	0.969-1.055			
LAD	0.700	1.019	0.927-1.120			
25(OH) Vitamin D	0.002	0.867	0.793 - .949	0.001	0.855	0.780-0.938
CPB time	0.742	1.003	0.984 -1.023			

25(OH) vitamin D = 25-hydroxy vitamin D; CPB=cardiopulmonary bypass; DM=diabetes mellitus; EF=ejection fraction; HT=hypertension; LAD=left atrium diameter; PoAF=postoperative atrial fibrillation

## DISCUSSION

In our study, the effect of the vitamin D levels was assessed on development of PoAF in patients who had isolated CABG. The association between lower vitamin D levels and PoAF development was determined. Plasma 25 (OH) vitamin D levels was found significantly lower in patients who developed PoAF than the patients with sinus rhythm. In univariate and multivariate logistic regression analysis, lower plasma 25 (OH) vitamin D levels and age > 65 years were found to be an independent variable predicting the development of PoAF. Also it was determined for cut-off level of 7.65 of vitamin D level for predicting PoAF with a sensitivity of 60% and a specificity of 64% in ROC analysis.

The incidence of PoAF following CABG surgery is seen in 25%-40% of cases. However, its frequency reaches to 62% following combined CABG and valve surgery^[^^7^^]^. The risk of PoAF in valvular and combined surgery, including coronary and valvular, was reported to be higher than that in coronary surgery alone. Mariscalco et al.^[^^8^^]^ identified the PoAF rates as 22.9%, 39.8%, and 45.2% for the isolated CABG, valve surgery, and combined surgery, respectively. Therefore, patients with valvular heart disease were excluded to not affect on the outcome of the study.

Postoperative hypoxemia was shown to be the most common cause of cardiac arrhythmias. COPD is an independent risk factor for arrhythmias, especially for AF and cardiovascular morbidity and mortality. In a large-scale, retrospective, case-control study, patients with COPD had a 4.41 times higher risk of AF and COPD is present in 10-15% of patients with AF^[^^9^^]^. Mathew et al.^[^^10^^]^ have showed that COPD increased the incidence of both persistent and paroxysmal AF and the incidence of PoAF increased to 43% in the presence of COPD. For this reason, patients with COPD were not included in order to make our results more accurate.

In majority of the studies, hypertension, diabetes mellitus, left atrium diameter and low ejection fraction were shown to play a role in the development of PoAF^[^^11^^]^. In our study, in terms of these parameters there were not significantly different between the groups. However, these factors which are effective in AF development are included in the logistic regression analysis. Any of these variables were not significantly associated with development of PoAF. These results may contribute to the effectiveness of the vitamin D level which measured for predicting PoAF in this study.

Age-related changes, including atrial fibrosis and accumulation of amyloid, can cause intraatrial reentry which leads to the development of AF^[^^12^^]^. Age has been repeatedly shown to be the major risk factor for AF after cardiac surgery^[^^13^^]^. In our study, significant difference was not observed between two groups in terms of age. But, in terms of in patients with age > 65 years was statistically significant difference between two groups. In our study, in binary logistic regression analysis, age > 65 years was found to be an independent variable predicting the development of PoAF. Cerit et al.^[^^14^^]^ found that age was significantly associated with development of PoAF following CABG in univariate logistic regression analysis. In another study, Geçmen et al.^[^^15^^]^ showed that age was as an independent variable predicting the development of PoAF in both univariate and multivariate logistic regression analyses. It was found in two different studies that search the relationship between vitamin D and AF in patients undergoing CABG that age was not significantly associated with development of PoAF following CABG^[^^16^^,^^17^^]^. Our results were generally consistent with the literature. When age is considered as a risk factor, it is known by researchers that elder patients have a risk for development AF.

The relationship between low 25(OH) vitamin D levels and AF has not been clarified yet. Several studies demonstrated a close association between vitamin D deficiency and AF. Demir et al.^[^^18^^]^ found that the relationship between vitamin D deficiency and non-valvular AF. Previous a study has shown that there is a relationship between low vitamin D levels and increased C-reactive protein levels^[^^19^^]^. The vitamin D has antioxidant properties that protect against oxidative stress in the atrium^[^^20^^]^. Zhang et al.^[^^21^^]^ in a meta-analysis suggested that there is a positive association between vitamin D deficiency and the risk of AF.

On the other hand, The Framingham Heart study, in which approximately 3000 patients were included and follow-up for approximately 10 years. Rienstra et al.^[^^22^^]^ found that vitamin D status is not an important contributor to AF in the general population. In a study in which they investigated postoperative AF, Skuladottir et al.^[^^23^^]^ found higher preoperative plasma 25(OH) vitamin D2 levels to be associated with an increased risk of POAF. In contrast, they did not observe an association between plasma levels of 25(OH) vitamin D3 or total 25(OH) vitamin D and the incidence of POAF. In another prospective cohort study based on the Rotterdam study, vitamin D level is not associated with AF^[^^5^^]^.

There are limited number of studies indicating the relationship between the vitamin D levels and PoAF in patients undergoing isolated CABG. In one of these studies, Gode et al.^[^^16^^]^ reported that 25(OH) vitamin D level were significantly lower in the PoAF group (*P*=0.007). In two similar studies, investigators found that there was no relationship between lower 25(OH) vitamin D levels and AF^[^^14^^,^^17^^]^. In our study, 25(OH) vitamin D level were significantly lower in the PoAF(+) group (*P*=0.002). Also, in our study, both in univariate logistic regression analysis and in multivariate logistic regression analysis, lower vitamin D levels were found to be as independent variables predicting the development of PoAF.

According to the US Endocrine Society guidelines, vitamin D deficiency was defined as 25(OH) vitamin D level less than 20 ng/mL, vitamin D insufficiency was defined as 25(OH) vitamin D level between 21 and 29 ng/mL^[^^24^^]^. The Institute of Medicine found that 25(OH) vitamin D serum levels of 16 ng/mL covers the requirements of approximately 50% of the population^[^^25^^]^. There are different opinions regarding the cut-off values for insufficient or deficient vitamin D level. In our study, 25(OH) vitamin D levels were as 12.13±7.98 ng/mL (range: 2.8-36.8), (percentiles 25; 6.67 ng/mL, percentiles 75; 15.35 ng/mL) in patients without PoAF. In patients with PoAF, 25(OH) vitamin D levels were found as 7.49±3.81 ng/mL (range: 1.5-20.5), (percentiles 25; 4.7 ng/mL, percentiles 75; 9.05 ng/mL). When these levels are taken into consideration, a vast majority of the patients in our study are in the vitamin D deficiency group according to the guidelines. It may be due to study period in the winter months. The average blood vitamin D level of the population studied is not clear. Although the blood vitamin D levels in most patients were insufficient according to the guidelines, the cut-off value that 7.65 ng/dl for vitamin D in this study was found to be statistically significant. Furthermore, it was determined a cut-off level of 7.65 for 25(OH) vitamin D level for predicting PoAF with a sensitivity of 60% and a specificity of 64% (AUC: 0.679, *P*=0.002).

In our study, the risk factors for arrhythmia were minimized and provided homogeneity between the groups; lower vitamin D levels would not affect the outcome of the study. It was more important for us that statistical differences in terms of vitamin D levels between groups are statistically significant predictors of postoperative AF development. On the other hand, our study had homogeneity. For this reason, the risk factors for development atrial fibrillation as COPD and valvular heart diseases were excluded. Therefore, the results of our study may be more specific in terms of the relationship between lower vitamin D levels and PoAF.

### Limitations of the Study

Our study has some limitations. The small sample size may be considered as the first limitation of this study, but the excess of exclusion criteria minimized the risk factors for PoAF. This may allow us to ignore the size of the sample, which appears to be a limitation of study. Secondly, parathyroid hormone levels were not measured. Also our work was only carried out in the winter and spring seasons. Further prospective studies with a larger number of patients are required.

## CONCLUSION

Many factors contribute to the development of AF after CABG. Many studies have been done on PoAF development. The lack of vitamin D can cause some of cardiovascular disease. In this study, lower vitamin D level was found to be an independent predictor of the development of PoAF. Low vitamin D level may be one of the reasons for PoAF development.

**Table:** 

Authors' roles & responsibilities	
KKÖ	Substantial contributions to the conception or design of the work; or the acquisition, analysis, or interpretation of data for the work; agreement to be accountable for all aspects of the work in ensuring that questions related to the accuracy or integrity of any part of the work are appropriately investigated and resolved; final approval of the version to be published
USS	Substantial contributions to the conception or design of the work; or the acquisition, analysis, or interpretation of data for the work; final approval of the version to be published
FT	Drafting the work or revising it critically for important intellectual content; final approval of the version to be published
NK	Drafting the work or revising it critically for important intellectual content; final approval of the version to be published
ŞY	Drafting the work or revising it critically for important intellectual content; agreement to be accountable for all aspects of the work in ensuring that questions related to the accuracy or integrity of any part of the work are appropriately investigated and resolved; final approval of the version to be published
